# The Department of Modern Nursing

**Published:** 1914-05-16

**Authors:** 


### THE DEPARTMENT OF MODERN NURSING.

### St. Bartholomew's Hospital.

There is an historical grandeur about St. Bartholomew's Hospital, dating as it does from the twelfth century, which colours the entire establishment. To walk round the magnificent collection of buildings, augmented by amalgamation with part of the Christ's Hospital premises, is to breathe the spirit of the past, and to admire the marriage of modern science with all that was most humane in the centuries which marked the rise of the great institution. The women who enter these portals to gain a knowledge of nursing should be lifted above personal ambitions and petty aims by the historic monuments and noble architecture among which their time is to be spent.

The hospital maintains 750 beds, seventy of which are at the Convalescent Home at Swanley. The average number occupied in the hospital is 567. The nursing staff consists of matron and assistant matron, two night superintendents, thirty-eight sisters, sixty-five nurses, 115 probationers in their second and third years, and from eighty-eight to ninety probationers in their first year.

### A Probationer's Admission and Course.

There is no preliminary school at St. Bartholomew's at present. But there is a preliminary examination which serves the double purpose of sifting out the best candidates, and of setting the women who wish to enter upon a course of training to prepare themselves beforehand for the lessons which await them. Slight as the knowledge of physiology and anatomy may be that is required, the papers set are calculated to reveal the candidate's capacity, and need not prove a bar to the admission of women who have shown ability without having yet attained to a very high standard of knowledge. Even a slight acquaintance with the subjects which are hereafter to be studied is a valuable asset for new probationers, and in our judgment some such preliminary home preparation might well be expected of all candidates for training. The written and oral examination is supplemented by a personal medical examination. The age limit is from twenty-three to thirty-three.

On being accepted as candidates probationers enter for two months on trial. Should their general conduct and abilities be considered satisfactory they are then appointed probationers, new admissions being made four times a year, at the beginning of February, May, August, and November. They provide for their own use all requisite uniform during the trial period. Lessons in Elementary Physiology and Anatomy are given during the first year by a member of the medical staff, and lessons in Practical Nursing by the Matron or by one of her assistants. The classes are held once a week at 8.30 P.M. A course of instruction in Bandaging is also given by one of the Matron's assistants. As a general rule probationers remain for three months at a time in a ward. Their instruction in nursing details rests with the Sisters of the ward, who are exceedingly keen in preparing their respective probationers for the tests to which they are subjected. Written reports are supplied to the Matron by the Sister of each ward relating to the conduct and proficiency of the probationers during the time spent in her ward. At the end of the first year the probationer is required to pass an examination, and should she fail in this her engagement with the hospital may be terminated. Those who pass this examination are appointed staff probationers. During the second year's training the lectures to be attended are on the nursing of Medical cases by a Physician, and on the nursing of Surgical cases by a Surgeon. No further examination has to be passed until the end of the third year, which concludes the training proper. During this third year they attend lectures on Elementary Bacteriology by the Pathologist of the Hospital, and on Gynæcological and Ophthalmic Nursing. They also attend classes given by the Sister in charge of the operating theatres. The final examination then takes place, and those who pass receive a certificate of efficiency. This certificate is not, however, handed to the nurse until the completion of a fourth year's service. A gold medal given by the Clothworkers' Company is presented to the probationer who passes first in the final examination, and a prize of books is also given by the same Company to the probationer who obtains the first place in the first year's examination.

Two courses are open to nurses after their certificate of competence has been obtained. They may become staff nurses in the hospital, or they may pass to the Trained Nurses' Institute and serve in their fourth year of service on the private nursing staff. As staff nurse there are many opportunities of adding to the knowledge of nursing and of obtaining wider experience.

### The Nurses' Quarters.

Being an ancient foundation, it is appropriate that the sisters should find quaint but ample quarters in the main blocks. So far as the nurses and probationers are concerned, in the modern sense their accommodation is yet to seek. Taken as a whole, anything more unsatisfactory or more condemnatory of the want of thoroughness and appreciation on the part of hospital authorities in regard to their responsibility to provide adequate accommodation for an army of young women working as nurses in a hospital is not to be found in any other hospital building of our acquaintance in the world. It is true, we believe, that for something like four decades at least the minutes have recorded that the most pressing need of this hospital is a nurses' home and adequate quarters for the nurses. Yet nothing is done in this direction, and the reason, we fancy, is to be found not so much with the governors as with the nursing staff. The present quarters are scattered all over the place, being mostly in small buildings formerly occupied as chambers by the students attached to the medical school, and under such conditions the possibility of exercising discipline and control must be slight indeed. The rabbit warrens plan, despite many defects, discomforts, and some anything but hygienic accommodation, does enable those who wish to, to escape to a great extent from discipline, and to do many things, if they want to, which would probably be impossible in a great nursing home, where adequate control and discipline could readily be enforced. The bath accommodation is sometimes more than defective. In one case an accurate and literal description of the bathroom would be "death-trap," and this particular unworthiness should be promptly scrapped.

The number of the sleeping rooms is inadequate; many are circumscribed and utterly unworthy of the traditions and importance of St. Bartholomew's Hospital. Why our oldest hospital should be the one institution which is unable to find a few men who are sufficiently interested in nurses and the credit of their great hospital to take hold of this matter and bring sufficient driving force to bear to supply an up-to-date nursing home for the staff, is one of those things which passes general comprehension. Why, too, with all the institutions in the country to choose from, probationers and the friends of probationers should ever consent to enter on training at St. Bartholomew's in the present condition of their sleeping and home accommodation, is another mystery that we should like to have solved by anyone, if such there be, who can explain it intelligently. The life of a probationer in a great hospital, under the most favourable conditions, and the demands upon her strength, are so onerous as to make the maintenance of sound health, often, anything but an easy matter for the young worker. Why, then, should a probationer or nurse consent to face avoidable difficulties and dangers—when they can avoid them without any loss of efficiency in training or any set-back in the reputation and standard which they may aspire to as trained and certificated nurses? One hundred thousand pounds is wanted at least to remove the grave structural and accommodation deficiencies at present prevailing at St. Bartholomew's Hospital. We should say that, if one or two of the younger governors with enterprise and knowledge were to take up this matter, publish an illustrated description of the quarters as they exist to-day, and show up all their anomalies with graphic directness, the requisite money would be forthcoming before the year was out. Many years ago the writer remembers being shown a report which recorded a large army of "Norfolk Howards," which had been captured in one of the beds in the nursing department. "Norfolk Howards" are now, we understand, far to seek, but the hygienic conditions and the structural defects are so gravely blameworthy and discreditable as to demand immediate attention at the hands of the governors with a view to the speedy application of an adequate remedy.

The authorities at St. Bartholomew's have had grave difficulties to contend with in adapting old buildings to serve the purposes of a modern Nurses' Home, and it cannot be said that these difficulties have been overcome. The nurses' quarters are in different blocks in the premises, and though in most respects their convenience has been studied and their comfort has been secured, it has not been found possible to provide a separate bedroom for each member of the nursing staff. It is, however, only the junior probationers who are called on to share a room with one other, and the cubicles are separated by a wooden partition or screen. If there are drawbacks to the bedroom accommodation, it must be observed that in respect to their refectory and general sitting-room the nurses at St. Bartholomew's are commodiously housed. The halls to which they have succeeded since the Christ's Hospital premises became available command satisfaction. Their beautiful proportions and historic associations render their use by the staff a real privilege, and from the material point of view no feature of comfort is lacking. The Sisters have private sitting-rooms adjoining their wards. There is a good tennis-court for the nurses' use. They also have the advantage of an admirable library. They pay 1s. a quarter as subscription to the reading-room, and thus secure an ample supply of books and periodicals.

### The Nurses' Health.

The health of the nursing staff is under the care of a physician specially appointed for that purpose. He comes to the Nurses' Home every morning and sees all nurses requiring attention. There are six comfortable sick-rooms with two beds in each, with sitting-room, bathroom, sink-room, etc., attached, for the use of sick nurses who have slight ailments. These are under the charge of the Matron's office assistant and a staff nurse. When the nurses are more seriously ill they are warded.

### Pay and Pensions.

The salaries for probationers are £8, £12, £20. In their fourth year they receive £30. If they remain on as staff nurses they receive £32 10s. for the fifth year and £35 for subsequent years. The Sisters' salary begins at £65 per annum, rising £6 10s. every five years to £84 10s., with lodgings, part board, washing, and uniform. No hospital in the kingdom equals St. Bartholomew's in liberality as regards pensions. Members of the nursing staff are eligible for pensions at fifty-five years of age, and they must retire at the age of sixty. By a recent resolution of the governors the voluntary retiring age has been reduced to fifty, but this decision was not made retrospective. In the case of ill-health, even if the required age has not been reached, they are eligible for a pension after ten years' service. The reproach which is sometimes levelled with some justice against the hospitals for using a woman's best years only to leave her without prospects is thus entirely obviated. The pension is based on one-fifty-fourth of the average salary and emoluments for the previous three years in respect of each year of service. The value of a Sister's emoluments is calculated at £60. The amount disbursed in pensions to the nursing staff in 1912 was £1,681.

### The Private Nursing Staff.

The Nursing Institute offers most favourable opportunities for work to nurses who have acquitted themselves well during their training and have the requisite qualities for private nursing. After the fourth year's service, which, as has been explained, may be spent either in the wards of the hospital or as private nurse at the Institute, nurses may apply to be taken on the permanent private staff under one of the two following schemes:

1. A salary of £30 a year, with a percentage on their earnings of 15 per cent., rising 5 per cent. each year to 30 per cent., with free board and residence in the Institute between cases, and a provision of indoor and outdoor uniform.

2. Full fees, less 7½ per cent. commission. When residing in the Institute nurses under this co-operative scheme pay 21s. per week for board and lodging, or at the rate of 3s. a day for shorter periods. The private staff varies in numbers from seventy to 100. The accounts are entirely separate from those of the hospital, and the nurses live under a different roof from the hospital staff, and are in no way connected with the hospital.

### Special Probationers.

Very good public service is carried on in the Special Probationers' Home in King Square, Goswell Road, situated about a mile from the hospital. Here ten or twelve ladies are received who desire to acquire some practical knowledge in nursing the sick by working in the wards of the hospital. They spend not less than three months in the Home, and pay thirteen guineas for each such period, the authorities reserving to themselves the right of terminating the engagement at any time and returning a proportion of the fee, should they think fit. The age of these probationers must be not less than twenty-one and not more than forty. They are required to discharge the same duties as those performed by regular probationers, and they attend all lectures and technical teaching given to members of the staff. After six months' work as special probationers they may, if considered suitable, join the regular staff. In this case the time spent as special probationers is allowed to count as part of the four years' training. This arrangement is not only calculated to be of the utmost service to women going out to the Colonies or in other ways likely to need an elementary knowledge of nursing, but it is also a very advantageous way of getting preparation for the duties of probationers. The special probationers have dinner and tea at the hospital, and all other meals at the King Square Home. Their hours are approximately the same as those of the regular probationers.

### Holidays and Leave.

In respect of holidays St. Bartholomew's is very liberal. Probationers are entitled to two weeks' holiday at the end of each six months of the first year of service—an excellent arrangement—and to four weeks at the end of the second and third years. They are allowed leave of absence for one whole day and one half-day every two weeks, and two hours off duty on five days in each week, together with "such leave of absence as can be arranged to suit the work of the various wards." Probationers are not kept on night duty for more than three months at a time, and during this time they are off duty, exclusive of meal-times, two hours a day. Nurses and probationers on night duty are allowed two consecutive nights off duty each month, when they may sleep away from the hospital if they wish.

